# Attentional Focus Strategies to Improve Motor Performance in Older Adults: A Systematic Review

**DOI:** 10.3390/ijerph20054047

**Published:** 2023-02-24

**Authors:** Ting Ting Chen, Toby C. T. Mak, Shamay S. M. Ng, Thomson W. L. Wong

**Affiliations:** Department of Rehabilitation Sciences, The Hong Kong Polytechnic University, Hong Kong SAR, China

**Keywords:** attentional focus, older adults, motor performance

## Abstract

Previous literature shows the beneficial effects of an external focus of attention on various sports skills in young adults. The objective of this systematic review is to evaluate the effects of external and internal focus of attention on motor performance in healthy older adults. The literature search was conducted in five electronic databases (PsycINFO, PubMed, SPORTDiscus, Scopus, and Web of Science). Eighteen studies that met the inclusion criteria were evaluated. Most of the motor tasks targeting older adults were related to postural control and gait. Over 60% of the included studies reported that the effect of an external focus was superior to that of an internal focus on motor performance in older adults. An external focus generally results in better motor performance than an internal focus among healthy older adults. However, the advantage of an external focus on locomotion may not be as significant as those illustrated in previous attentional focus studies. A challenging cognitive task may allow more automatic motor control than an external focus. Practitioners might provide clear instruction cues guiding performers to divert their attention away from their body and towards the movement effect for better performance, particularly in balancing tasks.

## 1. Introduction

### 1.1. Motor Performance in Older Adults

According to the latest report by the World Health Organization (WHO), the rate of population aging has accelerated dramatically in recent years. It is predicted that the total number of people aged over 60 will reach 2 billion in 2050 [1,2]. Motor performance of older adults, typically referring to balance [3], walking stability [4,5], posture stability [6], and movement speed [7], gradually deteriorates with the natural aging of the physiological system. This reduces the mobility required for daily activities in older adults and increases the risk of falls [8]. It was estimated that 30–40% of older adults over 65 fall at least once every year [9], and 20–30% of the older fallers suffer from moderate to severe injuries, which negatively affect their mobility and quality of life and increase healthcare costs [10,11]. To reduce deterioration and optimize motor functions, many studies have reported the benefits of various mobility-related strategies, such as resistance training [12,13], balance exercise [14], and Tai Chi [15,16], for motor performance in the older population. Providing individuals with different attentional focus instructions might also be an effective strategy to improve motor performance in older adults [17].

### 1.2. Attentional Focus Strategies

Previous research has observed that the attentional focus of an individual affects the performance of motor skills [18]. There are two types of attentional focus strategies: internal and external focus. Wulf et al. [19] were the first to define an “internal focus” as directing individuals’ focus to their body movements and an “external focus” as directing their focus to their movement effects on the environment (e.g., an apparatus or implement). Existing studies on attentional focus have generally recognized the benefits of adopting an external focus over an internal focus in motor learning and performance, such as golf [20], tennis [21], standing long jump [22], swimming [23], jump height [24], throwing [25], and striking combat sports [26]. To explain the mechanism of attentional focus, Wulf et al. proposed the constrained action hypothesis [27,28], positing that when the performer focuses on body movement (internal focus) and consciously controls the body to complete a motor task, the automatic control system that relies on an unconscious and spontaneous adjustment may be disturbed. On the other hand, when the performer focuses on the effects of the movement (e.g., on an apparatus) (external focus), it allows the automatic control system to control the movements, resulting in greater automaticity and more efficient movement patterns.

However, there are some controversies about the effectiveness of internal and external focus. For example, Wulf reported that the effect of external focus was limited for expert performers [29]. A possible explanation for this is that expert performers were so familiar with motor tasks that they spontaneously mobilized the automatic control system, even without explicit instruction [30]. Another study on juggling novices found that external focus may provide redundant information for sports tasks [31]. Yet, Ille et al. [32] observed that under external focus conditions, the reaction time and running time for sprints, both for expert and novice participants’, were significantly shorter than those under internal focus conditions. The conflicting results of the above studies indicate that the type of sports and the proficiency of the performers might be the potential confounders for the effects of attentional focus. Moreover, some other researchers have reported a lack of effect in their studies when targeting different populations (e.g., [33,34,35,36]). For example, the results of Emanuel et al. [36] were unclear about the effects on children when performing a dart-throwing task, whereas de Melker Worms et al. [35] showed no benefits in stability when older adults were given an external focus instruction during walking on a treadmill. These findings, therefore, raised questions about the reliability and generalizability to certain populations (e.g., children and older adults).

### 1.3. Objectives

While a few studies have systematically reviewed the benefits of attentional focus on specific motor tasks/performance (i.e., balancing and long jump) in the general population [37,38], the effects on specific groups of population, such as healthy older adults, remain unclear and warrant an investigation to synthesize the existing evidence. To the best of our knowledge, there is no systematic review that investigates how internal focus and external focus affect motor performance in older adults, a population that is more representative of clinical and rehabilitative application. Therefore, the main objective of this systematic review is to critically evaluate the effects of attentional focus strategies (external and internal focus) on motor performance in the population.

## 2. Methods

The protocol for the current review was prospectively registered with the International Prospective Register of Systematic Reviews (PROSPERO) (Registration ID: CRD42021241466).

### 2.1. Search Strategy

The literature search was conducted in February 2021 in five electronic databases (PsycINFO, PubMed, SPORTDiscus, Scopus, and Web of Science) as well as other sources (Google Scholar). The following keywords were used: “attentional focus OR attentional focusing OR focus of attention OR attentional control OR external focus OR internal focus OR internal attention OR external attention”, AND “older* adults* OR older* people OR elderly OR the old OR the aged OR old age”. After removing the duplicates, each study was initially screened by title and abstract. Thereafter, potential eligible studies with the full texts available were independently evaluated and examined by the authors based on the inclusion and exclusion criteria.

### 2.2. Inclusion and Exclusion Criteria

The inclusion criteria for the studies were: (1) healthy older adults aged 60 or above, without any major medical condition or cognitive impairment (e.g., stroke, Parkinson’s disease, Alzheimer’s disease, multiple sclerosis, schizophrenia, or dementia); (2) the focus of attention (external and internal) during motor performance was manipulated; (3) assessment of motor performance, defined to include fundamental movement skills (e.g., throwing, striking, and jumping), motor fitness (e.g., agility, muscle strength, and flexibility), and basic abilities (e.g., balance control, locomotion, and coordination) [39]. Studies were excluded if they were: (1) correlation studies or descriptive studies; (2) reviews, meta-analyses, study protocols, conference papers, or book chapters; (3) published in a language other than English; or (4) unpublished materials or articles not peer-reviewed.

### 2.3. Quality Assessment

The quality of the studies concerned was assessed using the Quality Index (QI), which was designed to assess the methodological quality of both randomized and non-randomized studies [40]. Since there appears to be no standardized quality assessment instrument for laboratory-based observational studies, this current systematic review adopted the QI and selected 12 relevant items with reference to a previous review of a similar nature (e.g., gaze behavior) [41]. The maximum score available was 12, as described in Table 1. Two reviewers performed the quality assessment independently and discussed any discrepancies until a consensus was reached. The adapted QI can be used to evaluate 4 domains of the study, viz., (1) reporting (6 items), (2) external validity (2 items), (3) internal validity-bias (2 items), and (4) internal validity-confounding/selection bias (2 items). The first six items are scored with “Yes” (1 point) or “No” (0 points), while the rest are scored with “Yes” (1 point), “No” (0 points), or “Unable to determine” (0 points). A higher score indicates higher quality. The sum of all item scores was calculated to state the quality of the study.

### 2.4. Data Extraction and Analysis

Eligible articles were screened independently by two review authors (T.T.C. and T.C.T.M.). The data extracted from each study for systematic evaluation were as follows: study characteristics, participant characteristics, group conditions, focus instructions, outcome measures, and main findings. From the extracted data, we performed a narrative synthesis that involved the use of textual description, grouping, and vote counting to summarize and produce a description of patterns across the included studies.

## 3. Results

### 3.1. Trial Flow

**Figure 1:**
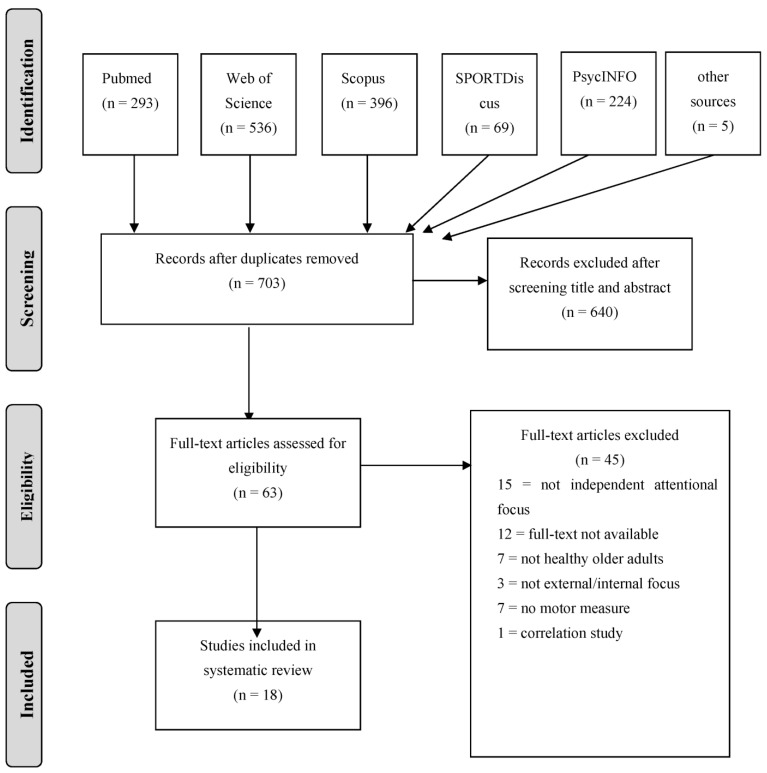
Preferred Reporting Items in Systematic Reviews and Meta-analyses (PRISMA) flow diagram for selecting the studies.

A total of 1518 studies were identified from five databases (PsycINFO, PubMed, ScienceDirect, Scopus, and Web of Science), and an additional five articles were identified from other sources. After removing duplicates, the remaining 703 studies were further screened by title and abstract, of which 63 were full-text analyses, and finally, 18 studies meeting the inclusion criteria were included in this systematic review. The details of the screening and literature selection process are presented in the Preferred Reporting Items in Systematic Reviews and Meta-analyses (PRISMA) flow diagram [42] (Figure 1).

### 3.2. Quality of Reviewed Studies

The maximum QI score of the reviewed studies was 12. Most studies had a relatively good quality score of 8 or more, while four scored 7 or less. As shown in Table 2, the four studies with relatively poor quality mainly lacked detailed reporting of participants’ characteristics and statistical output. The limitation of most of the reviewed studies was external validity, which was primarily due to the limited description of the sampling method or the adoption of convenience sampling, resulting in the reduced representativeness of these findings for the population.

### 3.3. Study Characteristics

The 18 studies included were published between 2009 and 2021 (Table 3). Two were published between 2009 and 2010, four between 2011 and 2015, and 12 within the past five years. Regarding study design, most studies were cross-sectional studies, with just two being randomized controlled trials (RCTs) [34,55] and six dividing subjects into either an internal focus group or an external focus group [34,44,45,46,51,55].

As for the group condition, the attentional focus strategy consisting of internal focus and external focus was the independent variable in all the included studies. Regarding the comparison groups or conditions, nine studies had a control group or condition with no instructions [33,44,47,48,49,50,52,54,56], and three studies included a dual-task condition with the concurrent cognitive task [52,53,54].

### 3.4. Participant Characteristics

A total of 768 older adults aged between 60 and 90 years were involved in this systematic review. All but two studies [17,33] had reported the number of males and females separately, and the male-female ratio was 0.65 [male (n) = 291; female (n) = 445]. All except three studies that did not specify participant characteristics [43,44,45] included participants with normal cognitive function and without any neurological, musculoskeletal, and/or cardiovascular impairments or other medical conditions limiting their daily activities.

### 3.5. Focus Instructions

In all studies, the external focus required participants to focus on the task targets (environmental effects), whereas participants with internal focus paid attention to body parts (body movements). Regarding the focus protocol, the duration of each trial in most studies was 30 s. As for intervention sessions, three of the included studies conducted five sessions [46], ten sessions (5 weeks) [34], and sixteen sessions (4 weeks) [33]. Only one of the included studies measured the effects of the retention phase [45].

### 3.6. Outcome Measures

The types of motor performance varied extensively among the included studies, including postural stability [17,33,43,52,53,54,55], balance [44,45], motor learning [34], walking stability [35,48,49,56], muscle power [46,50], walking efficiency [47], and sit-to-stand performance [51]. Among the 18 included studies, the stability of the anterior-posterior axis and the medial-lateral axis were the most commonly used measures of postural control, and time in balance and basic gait parameters were common measurement indicators for balance and gait, respectively.

### 3.7. Effects of Intervention

#### 3.7.1. Effects of Attentional Focus

Among the 18 studies included in this systematic review that compared external focus and internal focus, 11 reported that external focus resulted in better task-related outcomes of older adults, as opposed to an internal focus [17,43,44,45,47,48,49,50,52,54,55], six studies reported similar effects [33,34,35,46,53,56], and one reported an opposite effect [51] (Table 4).

Nine studies involved a control group or condition (e.g., with no explicit instruction). When comparing an external focus with a control group/condition, four of the studies reported better performance in external focus [33,44,52,54], while five of them reported a similar effect [47,48,49,50,56]. When comparing an internal focus with control, one reported better performance in internal focus [33], five of them reported a similar effect [44,47,52,54,56], while three found that it has harmful effects [48,49,50].

When comparing the effect of attentional focus with a cognitive dual-task condition, all three relevant studies reported better performance under the dual-task condition where participants were asked to count the number of times a target digit was presented (e.g., count the total number of 3) in an auditory sequence (e.g., ‘379’, ‘325’), compared to both attentional focus conditions [52,53,54].

#### 3.7.2. Effects on Motor Skill

The effect of attentional focus strategies varied among the types of motor skills. Of the ten studies focusing on postural stability/balance, seven reported better performance in external focus relative to internal focus [17,43,44,45,52,54,55], and the remaining three reported a similar effect [33,34,53]. For the five studies related to gait (i.e., stability or efficiency), three reported better effects of external focus relative to internal focus [47,48,49], whereas two reported a similar effect [35,56]. The one study that examined sit-to-stand performance [51] reported a better performance for internal focus relative to an external focus. Of the two studies on muscle power, one reported better performance in external focus relative to internal focus [50], and the other reported a similar effect [46].

## 4. Discussion

The purpose of this systematic review was to evaluate and compare the effects of internal and external focus on motor performance in older adults. A total of 18 studies that met the inclusion criteria were selected from the databases. The QI scores of the included studies were mostly 8–10 out of 12. Most of the studies were cross-sectional studies.

Eleven out of 18 included studies reported that the effect of external focus was superior to that of internal focus on motor performance in older adults. While a majority of studies in the existing literature investigated the effect of attentional focus among athletes and young adults, its generalizability across populations, specifically older adults, was not clearly known. The findings of this review support our current understanding of the mechanisms for the differential effect of attentional focus and suggest that the beneficial effect of external focus could, to some extent, be transferred to the healthy older adult population.

However, the type and difficulty of the motor tasks might influence the effect of attentional focus on performance [18,57]. The motor task of the reviewed studies focused mainly on posture and gait, a typical area of interest for the aging population. While our review reported that external focus results in better performance in postural control and gait than internal focus, the beneficial effect of an external focus relative to control was inconsistent, especially in natural walking tasks [47,48,49]. We posit that the advantage of explicit external focus instructions on locomotion may not be as significant as those illustrated in previous attentional focus studies. Malone and Bastian [58] argued that human locomotion is regarded as a “well-practiced” daily task that can be performed automatically and effectively without a conscious effort by healthy older adults, a condition that resembles the goal of an external focus (i.e., allowing unconscious or automatic processes to control the movements). On the contrary, when one attempts to adopt an internal focus, the automaticity of walking might be disrupted, and the natural locomotion is compromised, as shown in [47,48,49]. In other words, when performing a naturally developed skill such as locomotion, healthy older adults are more likely to adopt an attentional focus similar to an external instead of internal focus; hence an external focus manipulation did not improve (nor impair) natural walking performance in our review.

A certain degree of task complexity or novelty (i.e., the difficulty as a function of an individual’s capability) might be another determining factor for the effect of external focus to emerge. According to the constrained action hypothesis [27,28], when a motor task is challenging, focusing on the movement effect (external focus) might encourage older adults to unintentionally utilize motor systems they have previously developed through practicing other similar tasks. These motor systems are characterized by greater automaticity in movement control, which allows quicker and more frequent movement adjustments, thus reducing errors and improving overall performance. On the contrary, when an individual directs attention to their body movements (internal focus) or receives no explicit instructions (control), it might promote slower and more conscious control processes, which interfere with movement fluency and hamper performance [59]. However, if the motor task is not challenging, such as locomotion which is deemed well-practiced with less novelty, the individual would not be induced to intervene since he/she is content with the current motor processes. Thus, an external focus instruction would not be anticipated to elicit extra benefits. Unlike locomotion, when considering postural control or balancing skills that also go through natural development, the observed beneficial effect of an external focus on these tasks in our review might be a consequence of increased task complexity/difficulty [57]. Most of the previous research in the literature that demonstrated the significant effect of external focus often requires executing or learning relatively complex sports skills or uses novices with little to no experience with the particular task [18]. Similarly, in this review, most of the studies that investigated postural control involved relatively challenging tasks that required standing on foam or an unstable surface/platform (e.g., [44] or balancing tasks that were novel to the participants [17]) hence matching the theoretical framework of the constrained action hypothesis.

The effectiveness of an external focus relative to an internal focus might be susceptible to specific conditions and instructions [18]. Similar evidence was found in the included studies that investigated the older population. For instance, the beneficial effect of the external focus has been claimed to increase with the distance of the external marker [60]. Baniasadi et al. [33] and Richer et al. [53] found that the movement effect might not be easily distinguishable from the body movements due to a relatively close distance between the external marker and the internal body, which might lead to the overestimation of the internal effect and the relative decline of the external effect. Moreover, a clear environmental impact (movement effect) is crucial. For example, participants were only informed of external instructions to watch the screen while walking on a treadmill instead of specifying the goal, as in the study of de Melker Worms et al. [35], which may have reduced the compliance for focus instructions, leading to the insignificant findings. Another potential issue might be related to whether the instruction of the task matched the appropriate measurement method. For example, the task of Baniasadi et al. [33] was to stand on a mat with a glass of water in hand. The external focus instruction on the glass might provide sensory cues about whether the glass and therefore the body, has moved with respect to gravity, presumably translating into the attempt to reduce body sway. In contrast, the internal focus instruction on keeping the hand holding the glass still may be counterproductive for the balance task, as keeping the hand still rather than making small adjustments may be counterproductive to reducing body sway. The measurement approach of quantifying body sway only but not hand movement might fail to fairly judge the success of the internal focus condition. It is also important to note that the processing of other types of information (e.g., visual cue, sensory cue, etc.) should be ideally similar in both sets of instructions (internal and external) [18]. For instance, the lack of access to the same sensory cues as the external focus condition (i.e., the sound of a metronome) in the internal focus condition might confound the findings in Yogev-Seligmann et al. [56].

Regarding the comparison with cognitive dual-task conditions, the effects of external and internal focus were not superior to those of the dual tasks in which participants were typically required to count the times of occurrences of one or more target digits [52,53,54]. The purpose of a cognitive task is to transfer the participants’ attention away from a concurrent motor task under a dual-task condition, thus potentially reducing the conscious control of movements and adopting a more automatic control [61]. Richer et al. [54] indicated that both cognitive task and external focus conditions provide benefits in postural control, but their induced strategies are likely to be different. Specifically, their follow-up study [52] provided evidence of this postulation in healthy older adults deriving from several complementary measures associated with the frequency content of postural control and the complexity of sway. They suggest that the external focus and a relatively easy cognitive task could still provoke a shift towards more automatized and efficient control of posture, but to a less extent than a more challenging cognitive task [52]. After all, cognitive tasks involve more complex mental processes such as decision-making and working memory [62], as evidenced by the high error rate in the cognitive task performance in the studies of Richer et al. [54] and Richer and Lajoie [52] that reflect adequate difficulty to require greater attention. Since attention might still be allocated to a particular movement’s control component during an external focus task, a more challenging cognitive task presumably diverts individuals’ attention further away from postural control. It allows even more automatic control of postural sway than an external focus.

To explore the influence of age on the effects of attentional focus on motor performance, four studies compared the effects of attentional focus strategy on young and older adults [17,43,51,52]. The results found that, albeit both young and older adults benefit from an external focus strategy, young adults generally performed better in terms of overall task performance, as older adults were more susceptible to adverse effects with the increasing difficulty of the attention task. This may be attributable to changes in aging-related motor patterns. For instance, for postural adjustment, young people preferred ankle strategies, while older people adopted hip strategies more often [63], which is associated with poorer postural stability due to reduced ankle/foot flexibility. According to the general slowing hypothesis (GSH), aging not only causes cognitive deterioration but also afflicts the motor field [64]. Nevertheless, it is crucial for older adults to perform motor tasks with less energy consumption. Previous research reported that the external focus tends to recruit motor units more effectively and minimize the co-contraction between agonist and antagonist muscle groups, resulting in greater force and less oxygen consumption [65], while the instruction of internal focus may increase the load on working memory and hinder the coordination of muscles [66]. Therefore, the external focus seems to be more economical and promotes energy conservation.

There are some limitations to this systematic review. Only two RCTs were screened, which may lower the reliability of the conclusions. Second, it is hard to summarize any training protocol for attentional focus since most included studies were cross-sectional but not interventional trials. The intervention duration integrated among the remaining limited number of studies may be of little reference significance. Third, there was considerable heterogeneity relating to the methodologies (e.g., study designs, groups/conditions) and the diversity of outcomes measurements, which presents a challenge to perform a meta-analysis for the reviewed studies. Another limitation is the lack of retention tests among the reviewed studies (only one study examined the retention phase). Further research is recommended to investigate the effects of attentional focus on long-term improvements. Moreover, our results can be generalized only to healthy older adults. Previous studies that examined attentional focus on balance among older adults with Parkinson’s disease have reported a positive effect of external focus only on those patients with a history of falls [67,68]. These individuals, perhaps due to higher levels of anxiety and/or fear of falling, tend to be relatively more cautious and consciously control movement (reinvestment) to ensure safety [58,69]. As such, they might divert attention to the external environment to a greater extent when given external focus instructions. Last, it should be noted that there are likely to be studies unavailable for access or conducting in languages other than English which are not included in the present review, and hence, the results of this review might be influenced.

For practical implication, the current review provides a synthesis to assist future researchers and practitioners in designing and implementing instructions that can potentially benefit motor performance in the geriatric population. Instructions or feedbacks that emphasize individuals’ body movements are common in clinical settings associated with motor skill re-learning, physical therapy, and rehabilitation among geriatric patients [70]. In addition, older individuals tend to be relatively cautious and aware of their body movements when encountering novel and complex motor tasks, presumably due to higher anxiety and/or motivation to complete the task [59]. However, considering that the overall effect of an external focus results in better motor performance, especially in postural control than an internal focus among older adults in this review, it is recommended that practitioners provide clear instruction cues guiding performers to divert their attention away from their body and towards the movement effect for better performance, especially in tasks that involve balancing. Nevertheless, the beneficial effect of an external focus is not clearly known in the retention and transfer phases due to the limited evidence gathered from this review (i.e., only one study tested the effect of the retention phase [45], and none explored the transfer effect). Therefore, further investigation is warranted to examine the maintenance of the effects of attentional focus and transfer effects on motor performance in the aging population.

For future studies, some suggestions are listed below, derived from evidence in this review for exploring attentional focus strategy as an intervention method to improve the motor performance of older adults:

(1) More RCTs are needed to provide strong evidence for the effects of external focus in older adults.
(2) To ensure compliance with the focus instructions, objective measures are necessary to monitor the focus status. Previous studies have reported the use of electroencephalography (EEG) to measure the type and continuity of the focus condition. Ellmers et al. [71] indicated that EEG T3-Fz coherence could be considered a valid technique for assessing attentional focus, while Radio et al. [72] observed an association between the activation of external focus and lower alpha frequency. In addition, an fMRI study reported that external focus could activate more brain regions associated with vision and ventral streaming pathways, whereas internal focus was related to brain regions for motor control (i.e., cerebellum) [73].
(3) The protocol of the attentional focus strategy for older adults can be further adjusted and clarified by clearly distinguishing internal focus instructions from external focus instructions, ensuring sufficient difficulty of the motor task, and improving the duration and frequency of interventions.
(4) The role of the retention phase and the transfer phase can be further clarified.

## 5. Conclusions

Our findings cautiously support that an external focus results in better immediate learning of various motor tasks than an internal focus among healthy older adults. Yet, its beneficial effect relative to control appears to be minimal in locomotor tasks. We also discovered that challenging cognitive tasks might induce better performance than external focus strategies. Practitioners could consider providing clear instruction cues that guide performers to divert their attention away from their body for better performance, especially in tasks that involve balance control. In consideration of factors such as unclear instructions, incomplete intervention prescriptions, the difficulty of the focus task, and the type of motor performance, well-designed focus protocols and more RCTs are needed to form a confirmed conclusion on the effectiveness of an external focus in improving motor performance in the population.

## Figures and Tables

**Table 1:** Adapted Quality Index items.

Category	Item Number	Item
Reporting	1	Is the hypothesis/aim/objective of the study clearly described?
	2	Are the main outcomes to be measured clearly described in the Introduction or Methods section?
	3	Are the characteristics of the patients included in the study clearly described?
	4	Are the main findings of the study clearly described?
	5	Does the study provide estimates of the random variability in the data for the main outcomes?
	6	Have actual probability values been reported (e.g., 0.035 rather than <0.05) for the main outcomes except where the probability value is less than 0.001?
External validity	7	Were the subjects asked to participate in the study representative of the entire population from which they were recruited?
	8	Were those subjects who were prepared to participate representative of the entire population from which they were recruited?
Internal validity (bias)	9	Were the statistical tests used to assess the main outcomes appropriate?
	10	Were the main outcome measures used accurate (valid and reliable)?
Internal validity (confounding)	11	Were the patients in different intervention groups (trials and cohort studies), or were the cases and controls (case-control studies) recruited from the same population?
	12	Were study subjects in different intervention groups (trials and cohort studies) or were the cases and controls (case-control studies) recruited over the same period of time?

**Table 2:** Quality Index for Included Studies.

Studies	Reporting						External Validity		Internal Validity-Bias		Internal Validity-Confounding		Total Score
	1	2	3	4	5	6	7	8	9	10	11	12	
Aloraini et al. (2019) [43]	1	1	0	0	1	1	U	U	1	1	1	1	8
Ardakani et al. (2015) [44]	1	1	0	0	1	0	U	U	1	1	1	U	6
Baniasadi et al. (2018) [33]	1	1	1	0	1	0	U	U	1	1	1	1	8
Chiviacowsky et al. (2010) [45]	1	1	0	0	1	0	0	0	1	U	1	U	5
de Bruin et al. (2009) [34]	1	1	1	0	1	0	0	0	1	1	1	U	7
de Melker Worms et al. (2017) [35]	1	1	1	1	1	1	U	U	1	1	1	1	10
Hagh et al. (2013) [46]	1	1	1	0	1	1	0	0	1	1	1	U	8
Mak et al. (2019) [47]	1	1	1	0	1	1	0	0	1	1	1	1	9
Mak, Young, Chan, et al. (2020) [48]	1	1	1	0	1	1	0	0	1	1	1	1	9
Mak, Young, and Wong (2020) [49]	1	1	1	1	1	0	0	0	1	1	1	1	9
Makaruk et al. (2015) [50]	1	1	1	0	1	0	0	0	1	1	1	1	8
McNevin et al. (2013) [17]	1	1	1	0	1	1	0	0	1	1	1	1	9
Pinto et al. (2021) [51]	1	1	1	1	1	1	U	U	1	1	1	U	9
Richer and Lajoie (2020) [52]	1	1	1	0	1	0	U	U	1	1	1	1	8
Richer et al. (2017) [53]	1	1	1	1	1	0	U	U	1	1	1	1	9
Richer et al. (2020) [54]	1	1	1	0	1	0	U	U	1	1	1	1	8
Sangari et al. (2018) [55]	1	0	1	0	1	1	U	U	1	1	1	U	7
Yogev-Seligmann et al. (2017) [56]	1	1	1	1	1	1	0	0	1	1	1	1	10

Note: U—unable to determine. Please refer to Table 1 for detailed description of items 1–12.

**Table 3:** Summary of the Characteristics of the Reviewed Studies.

Studies	Study Design	Age [Mean (SD)]	Total N (M/F)	Group Conditions	Method/Focus Instructions	Outcome Measure
Aloraini et al. (2019) [43]	Cross-sectional, within-subject design	75 (5.85) 65+	10 (7/3)	External focus Internal focus (counterbalanced)	Optotrak 3D investigator Lower-extremity reaching task (Fitts’ task) Goal: be as fast and as accurate as possible in your pointing movement External focus: focus on the target Internal focus: focus on your foot Duration/Frequency: 108 × 10-s trial/condition	Postural adjustment movement time peak velocity time to peak velocity variability at target Anticipatory postural adjustments duration Anticipatory postural adjustments magnitude
Ardakani et al. (2015) [44]	Cross-sectional, between-subject design (randomized)	70.7 (2.6) 65–75	34 (34/0)	Group 1 (External focus): n = 17 age: 70.7 (2.4) Group 2 (Internal focus): n = 17 age: 69.4 (3.2)	MEGAWIN 6000 Balance tasks (normal standing, standing on foam, and standing on inflatable pillow) Goal: n.d. External focus: focus on the image that was installed on the opposite wall at 6-m distance. Internal focus: focus on leg muscles Duration/Frequency: 1 trial/task	Balance root mean square of data recorded from electromyography signal
Baniasadi et al. (2018) [33]	Pre-post, single-group, design	69.24 (5.77) 60–74	20 (n.d.)	Control (no instruction) External focus Internal focus (counterbalanced)	Biodex Stability System Postural task (stand on the mat and hold a glass of water) Goal: n.d. External focus: minimize movement of the glass over the duration of the trial Internal focus: minimize movement of the hand over the duration of the trial Training: planned model of proprioceptive exercises Duration/Frequency: 16 × 30-min session across 4 weeks	Postural sway overall stability index anterior-posterior stability index medial-lateral stability index
Chiviacowsky et al. (2010) [45]	Pre-post, between-subject design (non-randomized)	69.4 (6.57) 60–85	32 (8/24)	Group 1 (External focus): n = 16 Group 2 (Internal focus): n = 16	Stabilometer Balance training Goal: try to keep the platform as close to horizontal as possible External focus: keep the markers in front of feet horizontal Internal focus: keep feet horizontal Duration/Frequency: Practice phase: 10 × 30-s trial, 90 s rest between trials; Retention phase: one day later, 5 × 30-s trial, 90 s breaks between trials	Balance time in balance
de Bruin et al. (2009) [34]	Randomized controlled trial	81 (6) 70+	26 (21/5)	Group 1 (External focus): n = 12 age: 81.9 (6.8), M/F = 11/1 Group 2 (Internal focus): n = 14 age: 80.1 (5.4), M/F = 10/4	Biodex Stability System Balance training Goal: maintain dynamic postural stability on both stable and unstable surface conditionsExternal focus: focus on the air bubble in a level while shifting weight (with visual feedback screen) Internal focus: focus on belly while shifting weight (with visual feedback screen) Duration/Frequency: at least 3 practice trials/exercise, 25–35 min, twice a week, across 5 weeks	Motor learning weight shift score for medio-lateral movements performance time for the dynamic limits of stability test dynamic stability index timed get-up-and-go test time for 5 consecutive chair rises without the use of hands falls efficacy scale international questionnaire
de Melker Worms et al. (2017) [35]	Cross-sectional, within-subject design	69.3 (3.7) 65–78	28 (8/20)	External focus Internal focus (counterbalanced)	GRAIL system Walking task (5 min of treadmill walking including gait perturbations) Goal: preserve a stable locomotion pattern External focus: look ahead at the screen and concentrate on the movement of the treadmill belt Internal focus: look ahead at the screen and concentrate on the movement of legs Duration/Frequency: 5 min of walking/condition	Gait stability (means and coefficients of variation) step length step width stance time swing time
Hagh et al. (2013) [46]	Pre-post, between-subject design (non-randomized)	60–80	20 (10/10)	Group 1 (External focus) Group 2 (Internal focus)	Three-dimensional motion analysis system Walking training Goal: walk at self-determined pace along the walkway External focus: focus on markers along the side of the balance beam and take steps next to them Internal focus: focus on thigh’s moving forward and taking long strides Duration/Frequency: 5 sessions on different days, over 20 min/session	Sagittal ankle muscle power minimum powers maximum powers
Mak et al. (2019) [47]	Cross-sectional, within-subject design	70.3 (4.8)	134 (40/94)	External focus Internal focus Control (no instruction) (counterbalanced)	Electromyography Walking task Goal: walk at a self-selected pace along a 6 m walkway External focus: focus on the random series of digits ranging from 1 to 9 that will be presented on the computer monitor at destination when walking Internal focus: focus on lower limb movement when walking Duration/Frequency: 3 trials/condition	Walking efficiency Co-contraction of shank muscle groups Co-contraction of thigh muscle groups
Mak, Young, Chan, et al. (2020) [48]	Cross-sectional, within-subject design	70.3 (4.7) 65–90	140 (40/100)	External focus Internal focus Control (no instruction) (counterbalanced)	Three-dimensional motion analysis system Walking task Goal: walk at a self-selected pace along a 6 m walkway External focus: focus on the random series of digits ranging from 1 to 9 that will be presented on the computer monitor at destination during walking Internal focus: focus on body movements during walking Duration/Frequency: 3 trials/condition	Gait stability (variabilities) stride time stance time swing time percentage of double support time stride length step length step width sternum sway pelvis sway
Mak, Young, and Wong (2020) [49]	Cross-sectional, within-subject design	70.2 (4.8) 71.1 (4.8) 65+	76 (24/52)	External focus Internal focus Control (no instruction) (counterbalanced)	Three-dimensional motion analysis system Walking task Goal: walk at a self-selected pace along a 6-m walkway External focus: focus on the movement effect on external environment Internal focus: focus on body movements Duration/Frequency: 3 trials/condition	Gait pattern stride length step length step width stride time double support time stance time swing time gait speed sternum sway pelvis sway
Makaruk et al. (2015) [50]	Cross-sectional, within-subject design	64.8 (3.7) 60–69	23 (0/23)	External focus Internal focus Control (no instruction) (counterbalanced)	Monark cycle ergometer Cycling task Goal: maximum effort on the cycle ergometer External focus: focus on moving the pedals as fast as possible Internal focus: focus on moving legs as fast as possible Duration/Frequency: 1 × 10-s attempt/condition	Muscle power maximum power average work time to maximum power fatigue index
McNevin et al. (2013) [17]	Cross-sectional, within-subject design	70.80	12 (n.d.)	External focus Internal focus (counterbalanced)	AMTI force platform Postural task Goal: track a rotating light while maintaining an upright static posture External focus: keeping the tip of the stylus centered within the target Internal focus: keeping the knuckle of thumbs centered within the target Duration/Frequency: 6 × 30-s trial/condition	Postural Control time on target anterior-posterior and medial-lateral postural sway anterior-posterior sway
Pinto et al. (2021) [51]	Cross-sectional, between-subject design (counterbalanced)	68.84 (5.99)	57 (16/41)	Group 1 (External focus): 29 young, age: 23.72 (3.68), M/F = 7/22 27 older, age: 69.37 (6.46), M/F = 10/17 Group 2 (Internal focus): 30 young, age: 24.90 (3.26), M/F = 8/22 30 older, age: 68.37 (5.60), M/F = 6/24	Android-based application and sensors Mobility task (perform the sit-to-stand and stand-to-sit while holding a cup) Goal: (normal) perform the task as they usually do in daily life; (fast) perform the task as fast as they could without spilling liquid External focus: think all the time about the cup and the liquid inside the cup Internal focus: think about your own arm and the coordination of your movements Duration/Frequency: 3 blocks (3 difficulty levels) of 5 trials	Performance of Sit-to-Stand movement time inclination average inclination variability smoothness
Richer and Lajoie (2020) [52]	Cross-sectional, within-subject design	69.02 (3.47)	20 (15/5)	External focus Internal focus Cognitive tasks Control (no instruction) (randomized)	AMTI force platform Postural task Goal: stand quietly on the force platform with feet together and arms at their sides while looking at an eye-level target placed on a wall 3 m ahead External focus: minimize movements of the markers Internal focus: minimizing movements of the ankles Cognitive tasks: single-number sequence (SNS)-count the occurrence of a single digit; double-number sequence (DNS)-simultaneously counts the occurrence of two separate single digits Duration/Frequency: 4 × 60-s trial/condition	Postural control wavelet sample entropy rambling and trembling
Richer et al. (2017) [53]	Cross-sectional, within-subject design	71.9 (4.32)	16 (3/13)	External focus Internal focus Cognitive task (counterbalanced)	AMTI force platform Postural task Goal: stand quietly on the force platform with feet together and arms at their sides while looking at an eye-level target placed on a wall 3 m ahead External focus: minimizing movement of markers placed on the hips Internal focus: minimizing movement of the hips Cognitive task: silently count and sum the total occurrence of a preselected digit in a sequence of 3-digit numbers Duration/Frequency: 2 blocks of 3 × 60-s trial/condition	Postural stability area of 95% confidence ellipse deviation of the center of pressure in the anterior-posterior and medial-lateral directions velocity in the anterior-posterior and medial-lateral directions mean power frequency
Richer et al. (2020) [54]	Cross-sectional, within-subject design	69.2 (3.4)	20 (15/5)	External focus Internal focus Cognitive tasks Control (no instruction) (counterbalanced)	AMTI force platform and electromyography Postural task Goal: stand quietly on the force platform with feet together and arms at their sides while looking at an eye-level target placed on a wall 3 m ahead External focus: minimize movements of the markers Internal focus: minimizing the movements of their ankles Cognitive tasks: single-number sequence (SNS)—count the number of times a target digit was presented in the sequence and provide the total at the end of the trial. Double-number sequence (DNS)—count the number of times two target digits were presented in the sequence and provide two separate totals at the end of the trial Duration/Frequency: 4 × 60-s trial/condition	Postural stability area of 95% confidence ellipse standard deviation of center of pressure in the anterior-posterior and medial-lateral directions mean velocity in the anterior-posterior and medial-lateral directions Co-contraction index Tibialis anterior (TA) and Medial gastrocnemius (MG)
Sangari et al. (2018) [55]	Randomized controlled trial	72.50 (4.9)	80 (40/40)	Group 1 (External focus): n = 40 Group 2 (Internal focus): n = 40	Sensory Organization Test (SOT) balance master system Postural training Goal: n.d. External focus: identification of a signal Internal focus: counting backward from 100 to 3 Duration/Frequency: n.d.	Postural control the center of gravity alignment
Yogev-Seligmann et al. (2017) [56]	Cross-sectional, within-subject design	73.50 (6.41)	20 (10/10)	External focus Internal focus Control (no instruction) Dual-task (fixed order)	The GAITRite system Walking task Goal: walk at a normal comfortable pace on level ground along a well-lit obstacle-free walkway (15 m long) External focus: match steps to the rhythm of a metronome Internal focus: focus on keeping steps as consistent as possible Duration/Frequency: 8 trials/condition	Gait variability gait velocity average stride width average stride time average swing time average step length percentage of double support of the stride cycle time

Note: SD = standard deviation; N = number of participants; M/F = Male/Female; n.d. = not defined.

**Table 4:** Summary of the Results of the Reviewed Studies.

Studies	Attentional Focus Effect	Significant Main Findings
By comparisons of attentional focus		
EF > IF		
Aloraini et al. (2019) [43]	EF > IF	EF led to better postural adjustment when performing a lower extremity Fitts’ task than IF
Ardakani et al. (2015) [44]	EF > IF	EF reduced ankle muscle activity in conditions of standing on foam and standing inflatable cushions compared to IF
Chiviacowsky et al. (2010) [45]	EF > IF	EF was overall more effective in maintaining dynamic balance (time in balance) than IF
Mak et al. (2019) [47]	EF > IF	EF exhibited greater walking efficiency than IF
Mak, Young, Chan, et al. (2020) [48]	EF > IF	EF exhibited greater gait stability than IF
Mak, Young, and Wong (2020) [49]	EF > IF	EF exhibited more optimal gait pattern than IF
Makaruk et al. (2015) [50]	EF > IF	EF resulted in greater maximum muscle power than IF
McNevin et al. (2013) [17]	EF > IF	EF resulted in greater tracking accuracy under the suprapostural task than IF
Richer and Lajoie (2020) [52]	EF > IF	EF are better at promoting the automaticity of postural control than IF
Richer et al. (2020) [54]	EF > IF	EF led to greater postural stability than IF
Sangari et al. (2018) [55]	EF > IF	EF groups had a better postural control function than IF group
EF < IF		
Pinto et al. (2021) [51]	EF < IF	Worse angle stability (sit-to-stand performance) under EF compared to IF
EF = IF		
Richer et al. (2017) [53]	EF = IF	No significant differences in postural stability between EF and IF
Yogev-Seligmann et al. (2017) [56]	EF = IF	No significant differences in gait variability between EF and IF
Baniasadi et al. (2018) [33]	EF = IF	No significant differences in postural sway between EF and IF
de Bruin et al. (2009) [34]	EF = IF	No significant differences in weight shifting, dynamic balance, or functional abilities between EF and IF
de Melker Worms et al. (2017) [35]	EF = IF	No significant differences in gait stability between EF and IF
Hagh et al. (2013) [46]	EF = IF	No significant differences in ankle muscle power between EF and IF
EF/IF vs. Control		
Baniasadi et al. (2018) [33]	IF > Control EF > Control	Both EF and IF had better postural sway compared to Control
Ardakani et al. (2015) [44]	EF > Control IF = Control	EF reduced ankle muscle activity in conditions of standing on foam and standing on inflatable cushions compared to Control No significant differences in ankle muscle activity between IF and Control in three conditions
Mak et al. (2019) [47]	EF = Control IF = Control	No significant differences in walking efficiency between EF and Control and between IF and Control
Mak, Young, Chan, et al. (2020) [48]	EF = Control Control > IF	No significant differences in gait stability between EF and Control IF appear to compromise gait stability compared to Control
Mak, Young, and Wong (2020) [49]	EF = Control Control > IF	No significant differences in gait pattern between EF and Control IF appear to compromise gait pattern compared to Control
Makaruk et al. (2015) [50]	EF = Control Control > IF	No significant differences in maximum muscle power between EF and Control Control resulted in greater maximum muscle power than IF
Richer et al. (2020) [54]	Cognitive task&EF >Control Control = IF	Cognitive tasks and EF led to greater postural stability than Control No significant differences in postural stability between IF and Control No significant differences in co-contraction indices among all conditions
Richer and Lajoie (2020) [52]	Cognitive task&EF >Control Control = IF	Cognitive tasks and EF are better at promoting the automaticity of postural control than Control No significant differences in the automaticity of postural control between IF and Control
Yogev-Seligmann et al. (2017) [56]	EF = IF = Control	No significant differences in gait variability among EF, IF, and Control
EF/IF vs. Cognitive tasks		
Richer and Lajoie (2020) [52]	Cognitive task&EF>IF	Cognitive tasks and EF are better at promoting the automaticity of postural control than IF
Richer et al. (2017) [53]	Cognitive task > EF = IF	Cognitive task led to greater postural stability than both EF and IF
Richer et al. (2020) [54]	Cognitive task&EF >IF	Cognitive tasks and EF led to greater postural stability than IF No significant differences in co-contraction indices among all conditions
By motor skill type		
Postural stability/balance		
McNevin et al. (2013) [17]	EF > IF	EF resulted in greater tracking accuracy under the suprapostural task than IF
Baniasadi et al. (2018) [33]	EF = IF > Control	Both EF and IF had better postural sway compared to Control No significant differences in postural sway between EF and IF
de Bruin et al. (2009) [34]	EF = IF	Both EF and IF improved weight shifting, dynamic balance, and functional abilities No significant differences in any outcomes between EF and IF
Sangari et al. (2018) [55]	EF > IF	EF groups had a better postural control function than IF groups
Ardakani et al. (2015) [44]	EF > IF = Control	No significant differences in ankle muscle activity between IF and Control in three conditions EF reduced ankle muscle activity in conditions of standing on foam and standing on inflatable cushions compared to Control
Chiviacowsky et al. (2010) [45]	EF > IF	Both EF and IF improved their time in balance across practice trials and retention test EF was overall more effective in maintaining dynamic balance than IF
Richer et al. (2017) [53]	Cognitive task > EF = IF	No significant differences in postural stability between EF and IF Cognitive task led to greater postural stability than both EF and IF
Richer et al. (2020) [54]	Cognitive task&EF >IF&Control	No significant differences in co-contraction indices among all conditions Cognitive tasks and EF led to greater postural stability than IF and Control
Richer and Lajoie (2020) [52]	Cognitive task&EF >IF&Control	Cognitive tasks and EF are better at promoting the automaticity of postural control than IF and Control
Aloraini et al. (2019) [43]	EF > IF	EF led to better postural adjustment when performing a lower extremity Fitts’ task than IF
Gait		
de Melker Worms et al. (2017) [35]	EF = IF	No significant differences in gait stability between EF and IF
Mak et al. (2019) [47]	EF > IF EF = Control IF = Control	EF exhibited greater walking efficiency than IF No significant differences in walking efficiency between EF and Control and between IF and Control
Mak, Young, Chan, et al. (2020) [48]	EF = Control > IF	No significant differences in gait stability between EF and Control IF appear to compromise gait stability compared to Control
Mak, Young, and Wong (2020) [49]	EF = Control > IF	No significant differences in gait pattern between EF and Control IF appear to compromise gait pattern compared to Control
Yogev-Seligmann et al. (2017) [56]	EF = IF = Control = Dual-task	No significant differences in gait variability among EF, IF, Control and Dual-task
Sit-to-stand		
Pinto et al. (2021) [51]	EF < IF	Worse angle stability (sit-to-stand performance) under EF compared to IF
Muscle power		
Hagh et al. (2013) [46]	EF = IF	No significant differences in ankle muscle power between EF and IF
Makaruk et al. (2015) [50]	EF = Control > IF	EF and Control resulted in greater maximum muscle power than IF No significant differences in maximum muscle power between EF and Control

Note: EF = external focus; IF = internal focus.

## Data Availability

The data that support the findings of this study are available from the corresponding author upon reasonable request.
